# Supplementary figures and images for: Triple-Negative Breast Cancer Cells Recruit Neutrophils by Secreting TGF-β and CXCR2 Ligands
Source: Front Immunol. 2021 Apr 12;12:659996. doi: 10.3389/fimmu.2021.659996 (PMC8071875; doi:10.3389/fimmu.2021.659996)

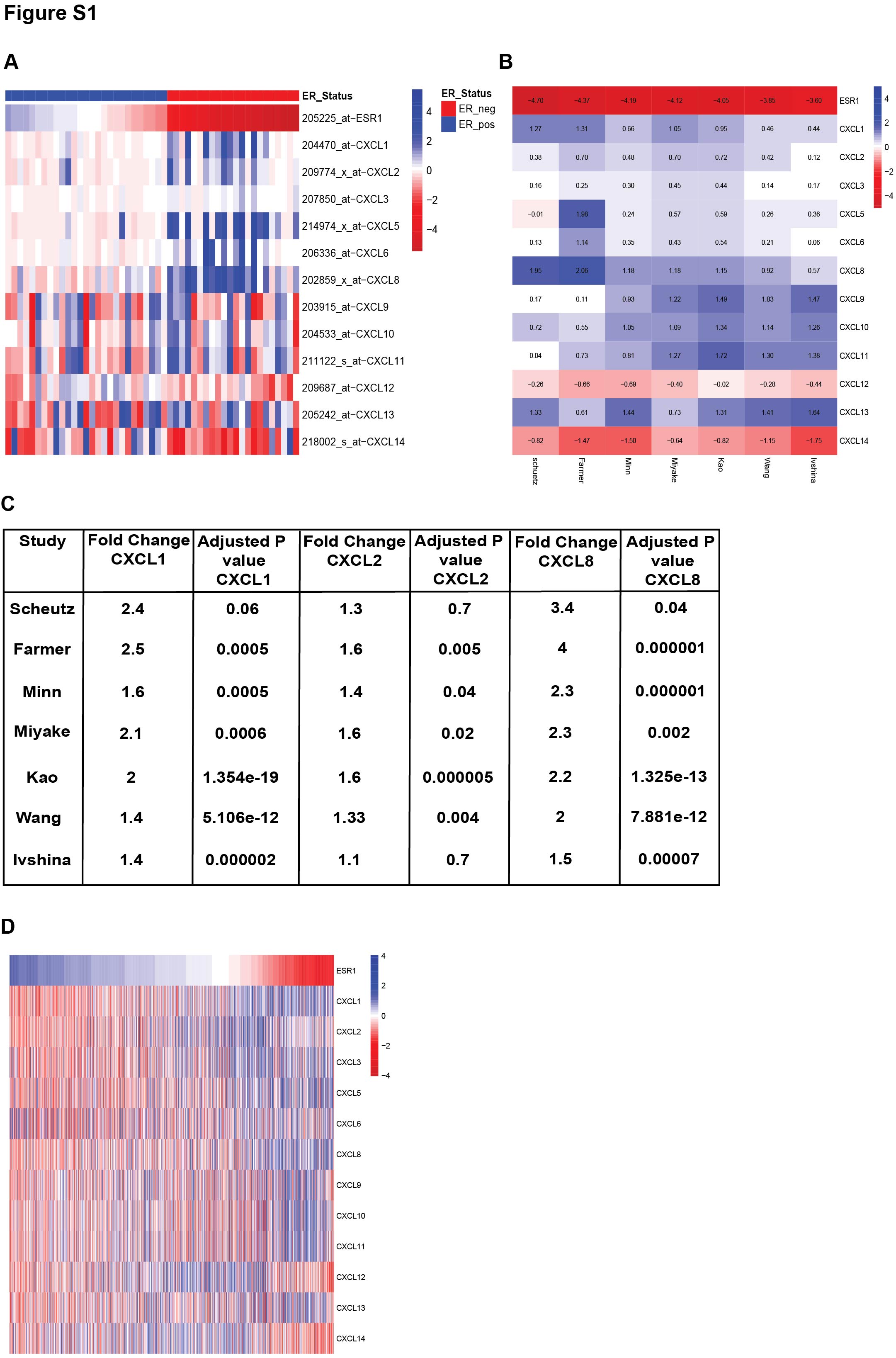

Supplement: Figure S1 — The expression of CXCL1, CXCL2, CXCL5, and CXCL8 is elevated in breast cancer specimens with low ESR1 levels. (A) Representative heatmap from the Farmer study [40] with ESR1 and CXCL chemokines annotated with specific probeset ID. Datasets identified from the Oncomine platform were used to compare CXCL transcript expression between ER+ (blue) and ER- (red) breast cancer specimens sorted by ESR1 expression levels. The heatmap was generated by plotting log2 fold change of expression values relative to averaged ER+ for ESR1 and each of the CXCL chemokines. Blue and red indicate high and low expression, respectively. (B) Heatmap generated by plotting log2 fold change of averaged ER- relative to averaged ER+ for ESR1 and each of the CXCL chemokines calculated from the datasets of N=7 independent studies (40–46). (C). Summary of fold changes for CXCL1, CXCL2, and CXCL8 between ER- and ER+ with the adjusted P value from each of the 7 studies. A fold change of 1.5 or greater and an adjusted P-value of 0.05 or less was considered statistically significant. (D). Datasets from TCGA-BRCA were analyzed for ESR1 and CXCL chemokine expression. The log2 of the FPKM values were plotted for the chemokines and ordered by the FPKM of ESR1. [file DataSheet_1.zip › Data Sheet 1/Figure S1.JPEG]

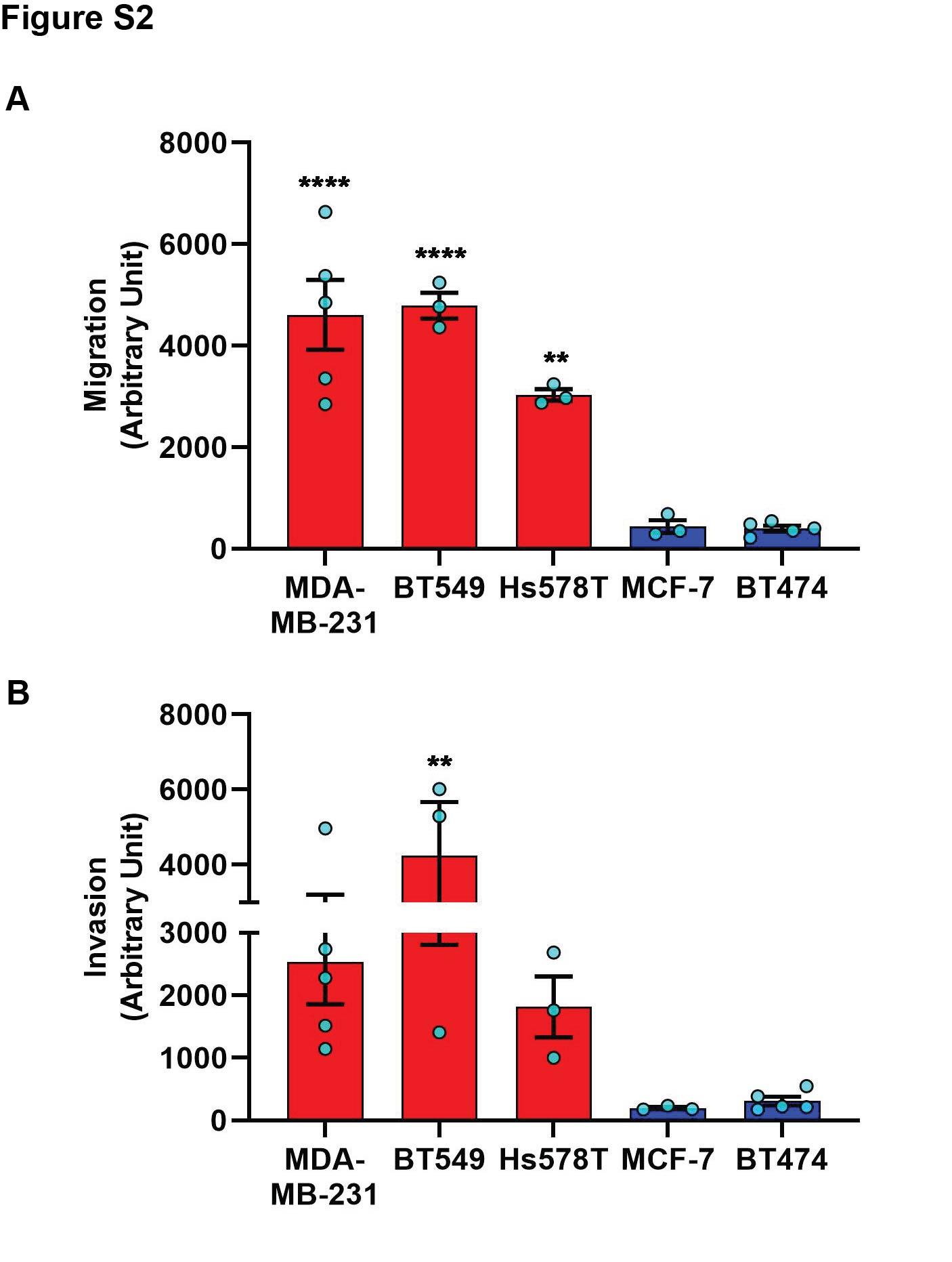

Supplement: Figure S1 — The expression of CXCL1, CXCL2, CXCL5, and CXCL8 is elevated in breast cancer specimens with low ESR1 levels. (A) Representative heatmap from the Farmer study [40] with ESR1 and CXCL chemokines annotated with specific probeset ID. Datasets identified from the Oncomine platform were used to compare CXCL transcript expression between ER+ (blue) and ER- (red) breast cancer specimens sorted by ESR1 expression levels. The heatmap was generated by plotting log2 fold change of expression values relative to averaged ER+ for ESR1 and each of the CXCL chemokines. Blue and red indicate high and low expression, respectively. (B) Heatmap generated by plotting log2 fold change of averaged ER- relative to averaged ER+ for ESR1 and each of the CXCL chemokines calculated from the datasets of N=7 independent studies (40–46). (C). Summary of fold changes for CXCL1, CXCL2, and CXCL8 between ER- and ER+ with the adjusted P value from each of the 7 studies. A fold change of 1.5 or greater and an adjusted P-value of 0.05 or less was considered statistically significant. (D). Datasets from TCGA-BRCA were analyzed for ESR1 and CXCL chemokine expression. The log2 of the FPKM values were plotted for the chemokines and ordered by the FPKM of ESR1. [file DataSheet_1.zip › Data Sheet 1/Figure S2.JPEG]

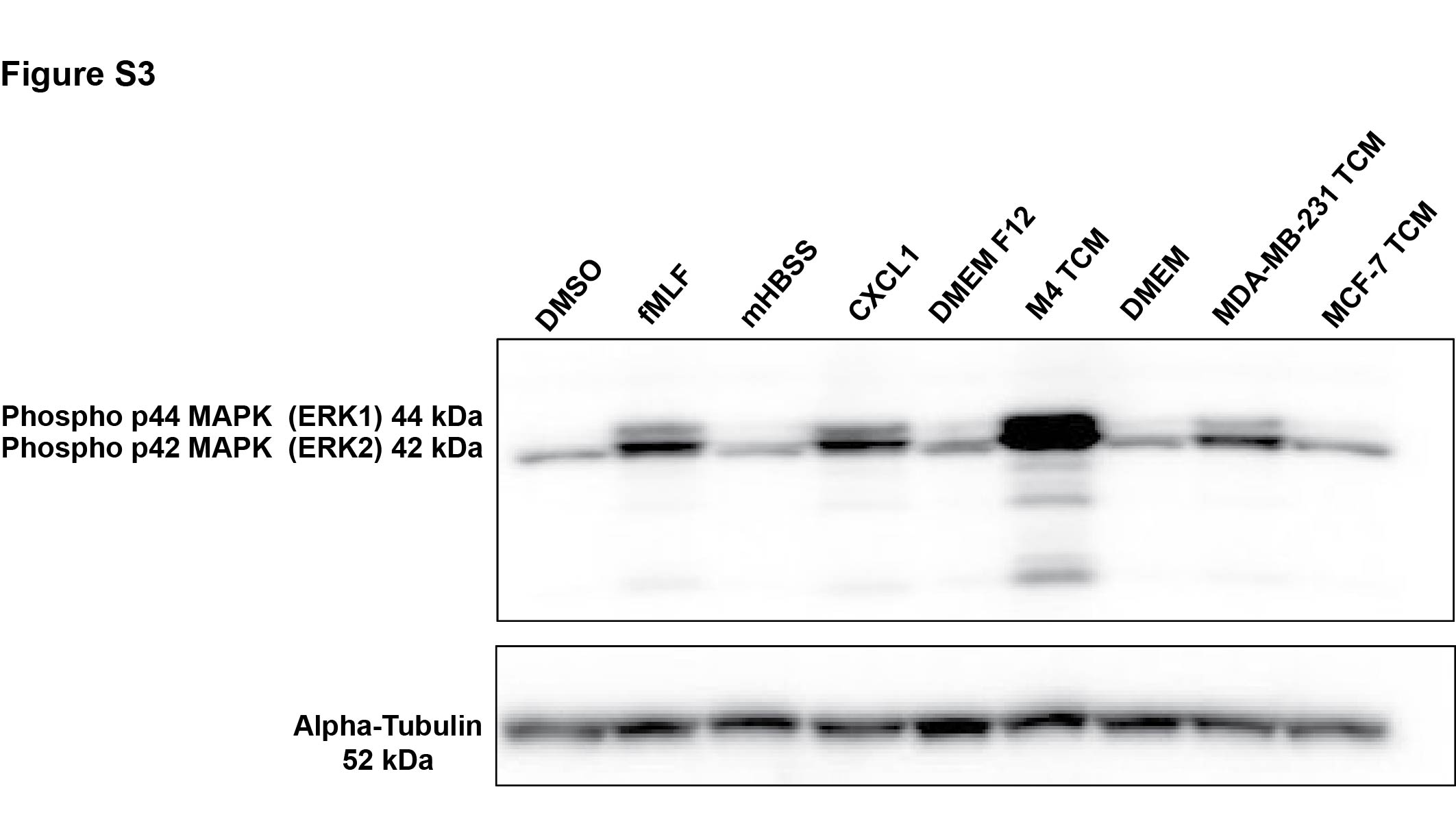

Supplement: Figure S1 — The expression of CXCL1, CXCL2, CXCL5, and CXCL8 is elevated in breast cancer specimens with low ESR1 levels. (A) Representative heatmap from the Farmer study [40] with ESR1 and CXCL chemokines annotated with specific probeset ID. Datasets identified from the Oncomine platform were used to compare CXCL transcript expression between ER+ (blue) and ER- (red) breast cancer specimens sorted by ESR1 expression levels. The heatmap was generated by plotting log2 fold change of expression values relative to averaged ER+ for ESR1 and each of the CXCL chemokines. Blue and red indicate high and low expression, respectively. (B) Heatmap generated by plotting log2 fold change of averaged ER- relative to averaged ER+ for ESR1 and each of the CXCL chemokines calculated from the datasets of N=7 independent studies (40–46). (C). Summary of fold changes for CXCL1, CXCL2, and CXCL8 between ER- and ER+ with the adjusted P value from each of the 7 studies. A fold change of 1.5 or greater and an adjusted P-value of 0.05 or less was considered statistically significant. (D). Datasets from TCGA-BRCA were analyzed for ESR1 and CXCL chemokine expression. The log2 of the FPKM values were plotted for the chemokines and ordered by the FPKM of ESR1. [file DataSheet_1.zip › Data Sheet 1/Figure S3.JPEG]

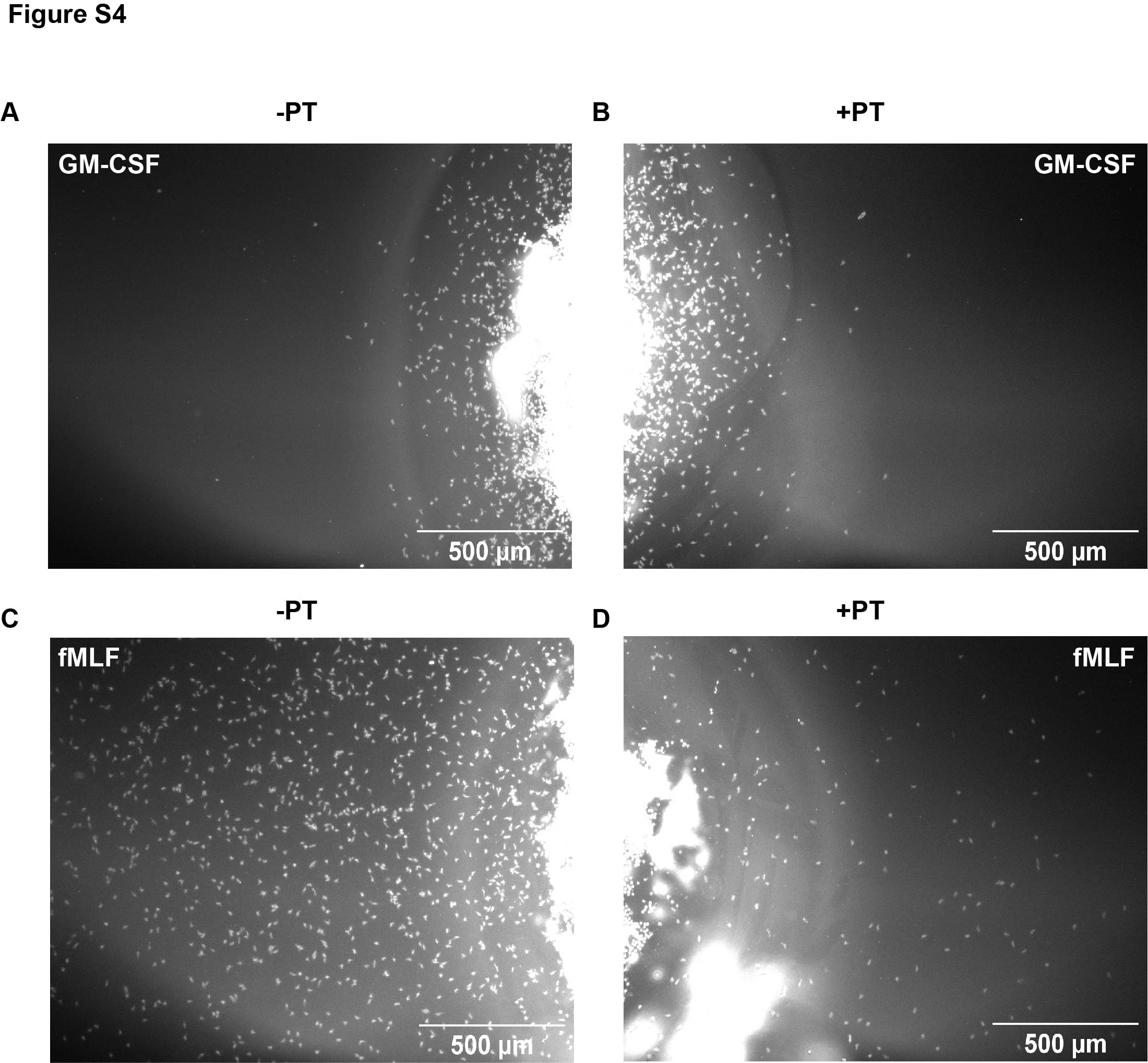

Supplement: Figure S1 — The expression of CXCL1, CXCL2, CXCL5, and CXCL8 is elevated in breast cancer specimens with low ESR1 levels. (A) Representative heatmap from the Farmer study [40] with ESR1 and CXCL chemokines annotated with specific probeset ID. Datasets identified from the Oncomine platform were used to compare CXCL transcript expression between ER+ (blue) and ER- (red) breast cancer specimens sorted by ESR1 expression levels. The heatmap was generated by plotting log2 fold change of expression values relative to averaged ER+ for ESR1 and each of the CXCL chemokines. Blue and red indicate high and low expression, respectively. (B) Heatmap generated by plotting log2 fold change of averaged ER- relative to averaged ER+ for ESR1 and each of the CXCL chemokines calculated from the datasets of N=7 independent studies (40–46). (C). Summary of fold changes for CXCL1, CXCL2, and CXCL8 between ER- and ER+ with the adjusted P value from each of the 7 studies. A fold change of 1.5 or greater and an adjusted P-value of 0.05 or less was considered statistically significant. (D). Datasets from TCGA-BRCA were analyzed for ESR1 and CXCL chemokine expression. The log2 of the FPKM values were plotted for the chemokines and ordered by the FPKM of ESR1. [file DataSheet_1.zip › Data Sheet 1/Figure S4.JPEG]

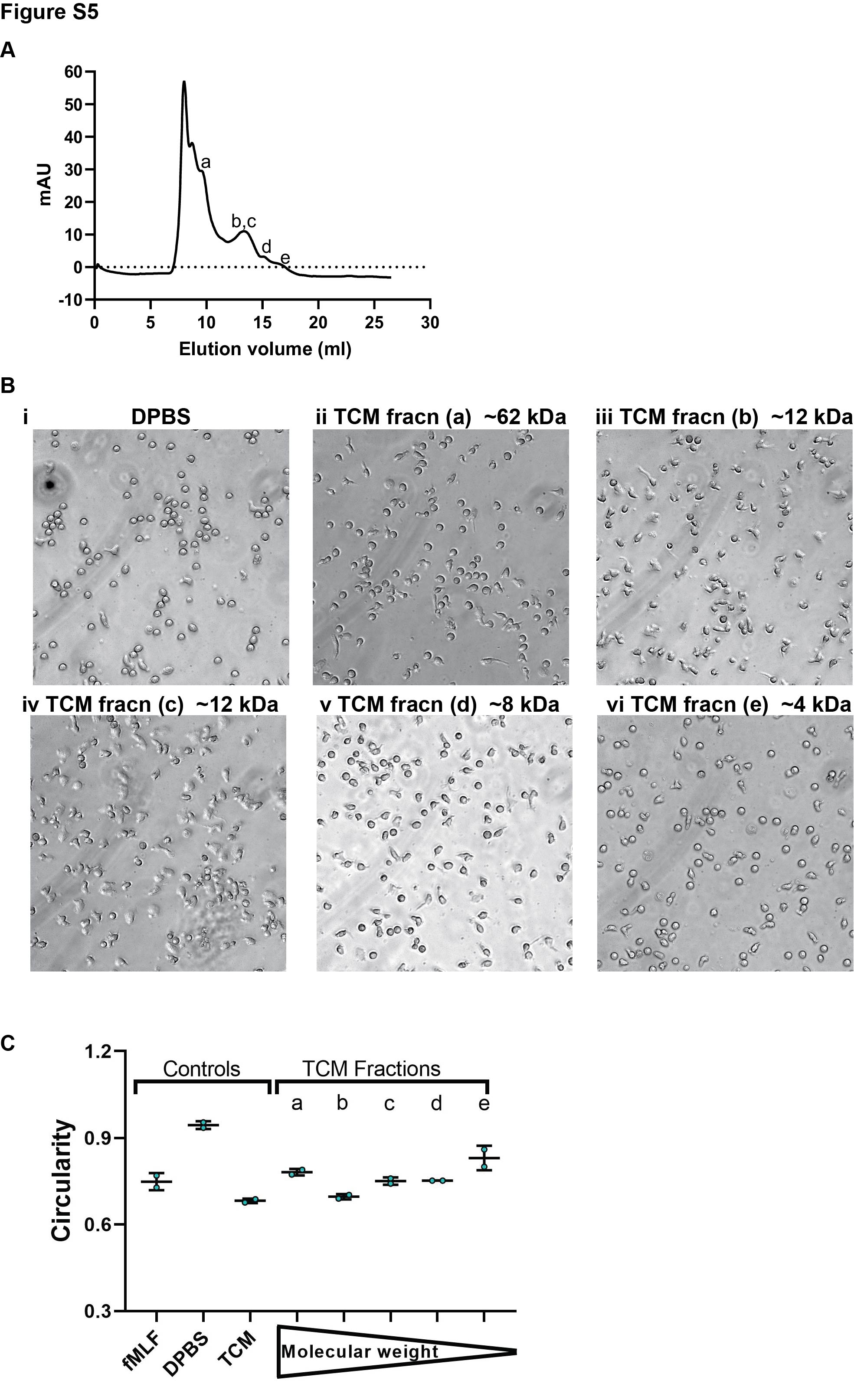

Supplement: Figure S1 — The expression of CXCL1, CXCL2, CXCL5, and CXCL8 is elevated in breast cancer specimens with low ESR1 levels. (A) Representative heatmap from the Farmer study [40] with ESR1 and CXCL chemokines annotated with specific probeset ID. Datasets identified from the Oncomine platform were used to compare CXCL transcript expression between ER+ (blue) and ER- (red) breast cancer specimens sorted by ESR1 expression levels. The heatmap was generated by plotting log2 fold change of expression values relative to averaged ER+ for ESR1 and each of the CXCL chemokines. Blue and red indicate high and low expression, respectively. (B) Heatmap generated by plotting log2 fold change of averaged ER- relative to averaged ER+ for ESR1 and each of the CXCL chemokines calculated from the datasets of N=7 independent studies (40–46). (C). Summary of fold changes for CXCL1, CXCL2, and CXCL8 between ER- and ER+ with the adjusted P value from each of the 7 studies. A fold change of 1.5 or greater and an adjusted P-value of 0.05 or less was considered statistically significant. (D). Datasets from TCGA-BRCA were analyzed for ESR1 and CXCL chemokine expression. The log2 of the FPKM values were plotted for the chemokines and ordered by the FPKM of ESR1. [file DataSheet_1.zip › Data Sheet 1/Figure S5.JPEG]
